# Effectiveness of pharmaceutical care for drug treatment adherence in patients with systemic lupus erythematosus in Rio de Janeiro, Brazil: study protocol for a randomized controlled trial

**DOI:** 10.1186/s13063-016-1317-1

**Published:** 2016-04-02

**Authors:** Marise Oliveira-Santos, José Fernando de Souza Verani, Luiz Antônio Bastos Camacho, Carlos Augusto Ferreira de Andrade, Rosele Ferrante-Silva, Evandro Mendes Klumb

**Affiliations:** Sergio Arouca National School of Public Health, Oswaldo Cruz Foundation, Rua Leopoldo Bulhões, 1480 Manguinhos, Rio de Janeiro Brazil; Pedro Ernesto University Hospital, State University of Rio de Janeiro, Boulevard 28 de Setembro n° 77, Vila Isabel, Rio de Janeiro Brazil; Evandro Chagas National Institute of Infectious Diseases, Oswaldo Cruz Foundation, Avenida Brasil n° 4.036, Manguinhos, Rio de Janeiro Brazil; Severino Sombra University, Av. Expedicionário Oswaldo de Almeida Ramos, n° 280, Centro, Vassouras, Rio de Janeiro Brazil; Lagoa Federal Hospital, Ministry of Health, Rua Jardim Botânico n° 501, Jardim Botânico, Rio de Janeiro Brazil

**Keywords:** Adherence, Pharmaceutical care, Systemic lupus erythematosus, Randomized clinical trial, Lupus nephritis

## Abstract

**Background:**

Treatment adherence is a primary determinant of the success and effectiveness of healthcare. Lack of adherence can lead to treatment failure and death. Although studies have shown that pharmaceutical intervention can improve drug treatment for patients with chronic diseases, studies on pharmaceutical care are not only inconsistent, they are scarce and limited to developed countries, include few patients, and are not studied in randomized clinical trials. Systemic lupus erythematosus is an autoimmune disease with high hospitalization and case-fatality rates. The adherence rate is low (31.7 %) in this group of patients in Brazil, and drug treatment for the disease is complex. Our objective is to evaluate the effectiveness of pharmaceutical care in drug treatment adherence in patients with systemic lupus erythematosus treated at a rheumatology outpatient clinic in Rio de Janeiro, Brazil.

**Methods:**

A randomized clinical trial (pragmatic trial) will be conducted. Adult participants (women) from a public hospital in Rio de Janeiro with a diagnosis of systemic lupus erythematosus will be followed for 12 months. A total of 120 patients will be randomized to two groups: intervention (Dader method for pharmaceutical care) and control (health/dietary counseling and risk reduction). The primary outcome will be drug treatment adherence evaluated by the eight-item Morisky Medication Adherence Scale. Secondary outcomes will be clinical improvement and quality of life.

**Discussion:**

Patients with systemic lupus erythematosus present with low treatment adherence, thus justifying the mobilization of human resources to optimize their clinical management. Despite the proven effectiveness of pharmaceutical care for various diseases, there are still no studies evaluating its effectiveness in systemic lupus erythematosus. Our hypothesis is that the intervention will also be effective in this patient group.

**Trial registration:**

ClinicalTrials.gov identifier: NCT02330250.

**Electronic supplementary material:**

The online version of this article (doi:10.1186/s13063-016-1317-1) contains supplementary material, which is available to authorized users.

## Background

Medicines are integral components of most therapeutic proposals, and lack of adherence is common in the treatment of chronic diseases. Inadequate compliance to the medical prescription can delay cure and hinder the control of a chronic disease [1–4]. A meta-analysis of 21 studies (46,847 patients) found that patients with low adherence had twice the mortality compared to those with good adherence [4]. Treatment adherence was also associated with better overall health status [4].

Key interventions to increase adherence can include educational (counseling on the medication and/or disease) and behavioral approaches, reinforcing the incorporation of therapeutic measures into the patient’s daily routine [5].

The inclusion of a pharmacist in the patient follow-up team allows the optimizing of drug treatment, preventing, detecting, and correcting problems with medication such as adverse reactions, interactions, and incompatibilities. Pharmaceutical care (PC), a practice focused on user care, has had a positive impact on health systems in various countries [6–14]. However, the range of research designs on PC needs to be expanded in order to allow its value as a professional practice to be proved.

PC is a practical proposal aimed at increasing understanding of the medical prescription and improving treatment adherence. This should help contribute to minimizing adverse drug reaction occurrence [15].

Various PC methods have been proposed, including the Pharmacotherapy Workup developed by Strand, Cipolle, and Morley [16] in the United States and the Dader method [17] of the Research Group for Pharmaceutical Care at the University of Granada, Spain.

Evaluating the effectiveness of a health intervention like PC requires analyzing the magnitude of the association between the cause (PC) and the effect (adherence). Obreli-Neto et al. [6] highlight that most evaluations of the effectiveness of drug treatment follow-up involve few patients and are not studied in randomized clinical trials. The few clinical trials focus mainly on conditions such as heart disease, dyslipidemias, and diabetes mellitus [4, 6, 18–25].

Systemic lupus erythematosus (SLE) is an autoimmune disease characterized by functional and/or anatomic biological disorders in multiple organs and systems [26]. Drug treatment for SLE is individualized, complex, long, and with an extensive dosing regimen. It is normally modified over time according to each episode of the active disease. Treatment is usually oriented towards the more severe forms, and various drugs are necessary [27]. Low adherence to drug therapy in SLE has been associated with increased mortality, besides leading to a significant economic burden [27–29].

Melchiors et al. [30] analyzed the systematic reviews published from 1990 to 2009 on the impact of pharmaceutical interventions on the clinical and economic outcomes of patients with various diseases. According to the 31 reviews with acceptable quality [30], rheumatic diseases, including SLE, have not been the focus of pharmaceutical interventions.

The implementation of PC in Brazil has been hindered by the absence of scientific documentation to provide evidence to health policymakers that PC is a valid investment [31]. A study in a university hospital in Rio de Janeiro showed that adverse drug reactions, absence of symptoms, and misunderstanding of the medical prescription can lead to patient non-adherence to treatment [2]. The current study’s objective is to evaluate the effectiveness of PC in adherence to drug therapy in patients with SLE, in controlling the disease clinically, and improving quality of life for treated individuals.

## Methods

### Study setting and subjects

The study population will consist of patients with a diagnosis of SLE according to the classification criteria of the American College of Rheumatology (ACR) [32] and the Systemic Lupus International Collaborating Clinics Group (SLICC) [33], followed at the rheumatology outpatient clinic of a public referral hospital for treatment of SLE in the city of Rio de Janeiro, Brazil. Appointments, laboratory tests, and treatment are offered free of cost to patients. The hospital’s rheumatology clinic is believed to provide superior care compared to that offered in Brazil’s public healthcare system as a whole. Even so, treatment adherence among patients with SLE in this hospital was only 31.7 % [2].

### Study design

An experimental design was considered the best methodological strategy to evaluate the effectiveness of PC, considering the need to control for numerous confounding factors, which are difficult to measure or poorly known. The design’s implementation attempted to preserve the routine conditions of care, so as to favor application of the study’s results to the health service’s real operational conditions (pragmatic trial).

### Inclusion and exclusion criteria

SLE has a broad spectrum of clinical manifestations and severity that influence the choice of drugs used [27]. To allow evaluation of adherence to drug therapy and its impact on disease activity, we opted to select a group of patients who were homogeneous in terms of clinical phenotype. Presence of nephritis defines a homogeneous profile of clinical manifestations and medication, so this group of patients was selected.

Patients will be included independently of treatment time. Only female patients will be included, because males comprise only 5 % of the total patient cohort. Preserving the same homogeneity and proportion of the sample, the reduced number of male patients would limit analysis of the sex variable.

Inclusion criteria:

- Diagnosis of SLE according to ACR [32] and SLICC [33] classification criteria
- Age 18 years or older
- Lupus nephritis evaluated by renal biopsy and histological classification of the International Society of Nephrology/Renal Pathology Society (ISN/RPS) 2003 [34], in classes III (focal proliferative glomerulonephritis), IV (diffuse proliferative glomerulonephritis), and/or V (membranous proliferative glomerulonephritis)
- Lupus nephritis without renal biopsy with acute renal failure or nephrotic syndrome secondary to SLE or with proteinuria ≥1 gram/24 hours or protein/creatinine ratio ≥1 in a single urine specimen
- Drug treatment that includes at least one specific drug for treatment of SLE in addition to corticosteroids, which are not specific but are frequently used to treat these patients

Exclusion criteria:

- Renal replacement therapy (dialysis)
- Renal transplant
- Use of intravenous immunosuppressive drugs
- Dependency on another person for administration of medication
- Psychiatric disease or cognitive impairment that prevents understanding the study’s questionnaires
- Pregnancy
- Refusal to sign the free and informed consent form
- Male patients

### Data collection and study procedures

Charts of all patients with appointments scheduled during the data collection period will be reviewed and evaluated by a rheumatologist and pharmacist, considering the eligibility criteria for participating in the study.

A pharmacist will explain the study’s objectives and invite the potential subjects. Those who agree to participate in the study and sign the informed consent form will be interviewed to answer structured questionnaires regarding socio-demographic data, lifestyle data, knowledge about SLE, and questions on current medication, to provide baseline descriptive data. Moreover, instruments to assess adherence to drug therapy [35], SLE activity [36], and quality of life [36–38], will provide information for the evaluation of the outcomes (further information in the “Evaluation of outcomes” section).

All patients will be randomized to one of the two groups for comparison: patients with PC and regular care according to the service’s routine (intervention) and patients who receive health and dietary counseling in addition to regular care (control).

### Randomization

Due to the importance of level of schooling for adherence [2] and for PC, we have attempted to maximize the groups’ similarity in this regard, using stratified randomization according to level of schooling: illiterate to incomplete middle school (stratum 1) and complete middle school to complete university (stratum 2).

Randomization was conducted by a researcher with no involvement in the fieldwork or clinical follow-up. Permuted-block randomization within subgroups of schooling allocated participants to intervention and control groups at a ratio of 1:1, using the WinPepi package (PEPI-for-Windows, version 11.32). The blocks’ size was set at six and was not disclosed to the study team.

Allocation of participants to intervention or control was concealed until the moment of applying the questionnaire. Assignment to intervention or control was kept in opaque envelopes to prevent seeing inside them, and the envelopes were sealed to prevent opening without tearing. The randomization lists containing the codes are sealed and will be kept secret until completion of data analysis.

Before sealing the envelopes, double verification was performed to confirm the sequential number and label pertaining to the selected group.

The envelopes were numbered from 1 to 72 (for each stratum) corresponding (on the randomization list) to one of the two study groups (intervention and control). Each envelope contained a card with adhesive labels with the participant’s number and allocation group, both printed, in order to identify each of the data collection instruments.

### Blinding

Due to the nature of the intervention with the patients’ follow-up being by the pharmacist and discussion of each clinical case being with the medical team, it is not possible to blind the intervention group from the volunteers and the field team. Nevertheless, dietary counseling in controls was meant to disguise intervention without interfering in the trial outcome. Blinding will only occur in the evaluation of the outcome.

### Study groups: intervention and control

The study participants (in the intervention and control groups) will be followed for 12 months starting on the date of inclusion in the study. They will receive the medical care routinely offered by the institution (centered on the medical team), which consists of clinical follow-up and counseling on the disease and its drug treatment. The number of appointments per patient will not be predefined in both groups, but will be determined by the patient’s attending rheumatologist, based on clinical assessment and SLE treatment guidelines [39–41].

In addition to the care offered by the institution, patients assigned to the intervention group will receive counseling by the pharmacist, referred to in this study as PC. This will be based on the Dader methodology [17] and will include data collection, identification of problems related to the medication, and implementation of a plan for patient care and follow-up.

The forms from the Dader method were digitized and had their layout adapted, with the inclusion of mandatory clinical and laboratory parameters for defining SLE disease activity, i.e., complete blood count, urine test, serum complement level, anti-DNA antibody titer, and protein/creatinine ratio. The textual content and application will follow the original Dader method [17].

The intervention (PC) will consist of individual patient follow-up in the rheumatology outpatient clinic. Appointments with the pharmacist will take place on the same day, following the medical appointment (Table 1).

**Table 1:** Steps in the follow-up of the intervention group based on the Dader method

Intervention group						
	Baseline data collection	Identification of critical points	Health education	Evaluation of prescription	Intervention	Final considerations
Baseline appointment	1. Socio-demographic data	1. Patients’ knowledge on SLE^a^ and drug treatment	1. Clarify queries on SLE, treatment, and medication	1. Identification of problems with medication	1. Counseling on the prescribed treatment regimen	1. Information on tests for next appointment
	2. Disease activity and clinical manifestations	2. Treatment adherence	2. Education on use and proper storage of drugs		2. Adjustment of drug dosing schedule to fit patient’s routine	2. Annotations on the patient’s medication chart
	3. Current medication		3. Counseling on possible adverse events from drugs		3. Counseling on the indication and use of each prescribed drug	3. Scheduling next appointment
	4. Quality of life		4. Distribution of information leaflets on SLE and drugs		4. Discussion with the medical team, if necessary, to adjust the prescribed medication	4. Setting treatment adherence targets
	5. Drug treatment adherence		5. Counseling on lifestyle changes, use of sunscreen, and healthy eating		5. Counseling on how to acquire the prescribed drugs	
Subsequent appointments		1. Analysis of laboratory tests	1. Clarify any remaining queries on SLE, treatment, and medication	1. Identification of problems with medication	1. Review of drug treatment	1. Information on tests for next appointment
		2. Medical team’s report on the consultation, impressions concerning the patient and disease course	2. Counseling on continuity of treatment, correct use of medication, and healthy habits		2. Solving of current or potential problems with drug treatment	2. Annotations on the patient’s medication chart
		3. Patients’ perceptions of their clinical status			3. Discussion with medical team on critical points observed in prescription, if any	3. Scheduling next appointment
		4. Treatment adherence			4. Discussion with the medical team, if necessary, to adjust the prescribed medication	4. Setting treatment adherence targets
					5. Adjustment of drug dosing schedule to fit patient’s routine	
					6. Counseling on how to acquire the prescribed drugs	

^a^SLE: systemic lupus erythematosus

Patients assigned to the control group will receive the medical care normally provided by the institution and follow-up by a non-pharmaceutical professional who will provide health and dietary and risk reduction counseling (Table 2). The information on their medication will continue to be provided by the attending physician as usual.

**Table 2:** Steps in follow-up of the control group

Control group			
	Baseline data collection	Health education	Final considerations
Baseline appointment	1. Socio-demographic data	1. Clarify queries on SLE^a^	1. Information on tests for next appointment
	2. Disease activity and clinical manifestations	2. Counseling on lifestyle changes, use of sunscreen, and healthy eating	2. Annotations on the patient’s medication chart
	3. Current medication	3. Distribution of information leaflets on SLE	3. Schedule next appointment
	4. Quality of life		
	5. Drug therapy adherence		
Subsequent appointments		1. Clarify any remaining queries on SLE, treatment, and medication	1. Information on tests for next appointment
		2. Counseling on healthy habits	2. Annotations on the patient’s medication chart
			3. Schedule next appointment

^a^SLE: systemic lupus erythematosus

**Fig. 1:**
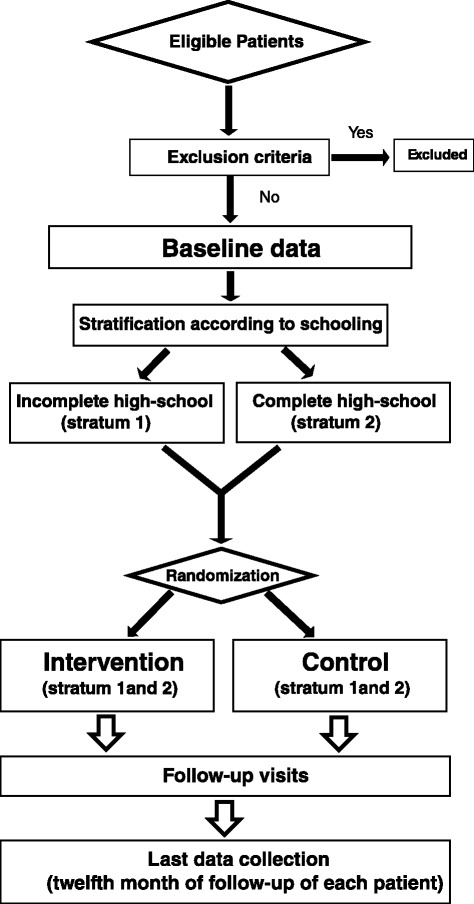
Study flow chart – flow diagram of the study design: stages of care delivery and follow-up of patients in the intervention and control groups

After 12 months of follow-up, participants in both groups will answer the same questionnaires used during the initial visit for evaluation of adherence to drug therapy, disease activity, and quality of life. The flow diagram (Fig. 1) briefly describes the stages of care delivery and follow-up of patients in the intervention and control groups.

### Fieldwork team

The team will consist of a research coordinator/supervisor (a pharmacist with a Master’s degree in epidemiology), a pharmacist who will follow the intervention group, a non-pharmacist professional who will work with the control group, a rheumatologist with a PhD in medical sciences and a Master’s degree in nephrology who will supervise the clinical follow-up, and an undergraduate pharmacy student who will assist in the fieldwork and keying-in of the study data.

All the team members have undergone a training program consisting of a review of basics on SLE diagnosis, treatment, and treatment objectives, discussion of clinical cases, and training in the Dader method [17].

The field team will follow a procedures manual with all the activities to be conducted with the patients and health team. The activities will serve as the basis for the data collection and attitudes to be adopted in the various stages of the study. The manual’s instructions will be followed closely to ensure standardization of procedures and minimal occurrence of errors.

A pilot study (eight patients not included in the larger study) was conducted aiming at assessing understanding of questions by the patients, increasing familiarity of the study team with data collection forms, and measuring the length of the interview. As a result, minor changes were made in the collection form on medication, and “fine tuning” of the field work.

### Evaluation of outcomes

The primary outcome will be drug treatment adherence (DTA). There is no “gold standard” for evaluating DTA. In this study, we chose to use the eight-item Morisky Medication Adherence Scale (MMAS-8) [42], since it encompasses the essential aspects of adherence to treatment, and has a translated and validated version in Brazilian Portuguese [35]. The eight questions have dichotomous answers (yes/no), formulated to avoid positive response bias by patients, using inversion of responses related to the interviewee’s adherence behavior. Although MMAS-8 performed well in a previous study on hypertension [42], we considered it appropriate to measure its reliability in SLE patients.

Based on MMAS-8, patients’ adherence will be classified as high (8 points), medium (6 to <8 points), and low (<6 points). DTA will be measured as the mean of the scores at two moments on the same day (test and retest for reliability assessment) during the baseline interview and after the pharmaceutical intervention or control.

Secondary outcomes will be disease activity and quality of life. Disease activity will be assessed by the Safety of Estrogens in Lupus Erythematosus National Assessment/Systemic Lupus Erythematosus Disease Activity Index (SELENA/SLEDAI) [36]: based on the score, patients will be classified as having inactive SLE (score of 0); mild activity (score of 1 to 5); moderate activity (6 to 10); high activity (11 to 19); or very high activity (≥20) [43]. Patients’ quality of life will be measured by the Medical Outcomes Study 36-Item Short-Form Health Survey (SF-36) [36–38], evaluating the following domains: functional capacity, physical appearance, pain, overall health status, vitality, social aspects, emotional aspects, and mental health. Of the different questionnaires used to evaluate quality of life in patients with SLE, only the SF-36 has been translated and validated in Brazilian Portuguese. It is the most extensively used instrument in studies that include patients with SLE [37, 38].

Medicines will be classified according to the Anatomical Therapeutic Chemical Classification System (ATC) elaborated by the Nordic Council on Medicines and recommended by the Drug Utilization Research Group (DURG) of the World Health Organization (WHO) for drug utilization studies (DUS) [44]. The ATC was developed as a uniform international classification of therapeutic drugs. WHO recommends the system, which allows comparison of drug utilization, and is highly useful for performing studies in pharmacoepidemiology [44].

### Sample size calculation

The study was designed to test the null hypothesis (H_0_) of no difference in DTA between the two study groups.

The number of participants was estimated based on expected 30 % adherence without the intervention [2] and a clinically relevant increment of 30 percentage points, expected with PC. The number of patients in each group was calculated to detect a 30 % difference in adherence to prescribed medication, with two-sided alpha of 0.05 and 80 % power.

To date there are no studies evaluating the effectiveness of pharmaceutical care in medication adherence in SLE. The 30-percentage-point difference was arbitrarily defined by rheumatologists from the service as a feasible and clinically relevant magnitude of the effect attributable to the intervention. That difference means the doubling of current adherence levels at the outpatient clinic, and also exceeds WHO estimates of adherence (50 %). The sample size calculated for each group was 48 patients, using EpiInfo Stat Calc7. An additional 20 % was added to compensate for possible losses due to dropout or death, resulting in a final sample of 116, rounded up to 120 patients, to account for block size.

### Statistical analysis

For both groups, at each visit, patients’ relevant data will be digitized in an electronic file with double data entry by two different entry clerks.

Data will be processed in the R Statistical Package [45] version R 3.2.2 using descriptive and analytical statistics. Analysis will be performed both with the “intention-to-treat” approach (includes all participants in their originally randomized groups) and in the subset that adhere to the study protocol (“per protocol analysis”). Reasons for loss to follow-up will be disclosed according to the Consolidated Standards of Reporting Trials (CONSORT) flowchart format (non-pharmacologic treatment interventions) [46], and analyzed accordingly.

Evaluation of the effectiveness of PC in DTA will be based on outcomes assessed by medication adherence between both groups at study end and, secondarily, measured adherence differences between baseline and end of follow-up within both groups. To adjust for multiple testing we will apply the Bonferroni correction [47].

Statistical data analysis, modeling, and interpretation of the results will follow the conceptual theoretical model used by Oliveira-Santos et al. [2] for hierarchical analysis of DTA in patients with SLE. Briefly, the study variables in this model are divided into hierarchical blocks: clinical aspects of SLE (proximal variables) establish the treatment options independently of the patient’s socio-demographic characteristics (distal variables), and health professionals/institutions act as intermediaries in the patient’s relationship to treatment (intermediate variables) (Table 3).

**Table 3:** Variables’ descriptors and hierarchical levels

Proximal	Intermediate	Distal
Lupus-related	Medical follow-up	Demographic, social and economic characteristics
Age at onset of symptoms		Schooling
Age at diagnosis	Missing scheduled appointment	Age
Disease duration		Marital status
Number of hospital admissions		Ethnicity
		Paid occupation
Clinical manifestations		Lupus as the cause of impairment
SELENA-SLEDAI		Total family income
Total score		Per capita family income
Drug therapy		Number of household individuals
Description		Drug disposal
Pharmacological group		Family support
Number of drugs		Individual patient-related factors
Dosage/day		Disease knowledge
		Symptoms
Over-the-counter (OTC) medicines		Chronicity
Drug-related adverse events		Treatment
Attitude toward side effects		Food habits
		Number of meals a day
		Food restriction or diet
		Alcohol intake
		Smoking
		Frequency (cigarettes/day)
		Years of smoking
		Physical activity
		Description
		Frequency (days/week)

*SELENA-SLEDAI* Safety of Estrogens in Lupus Erythematosus National Assessment/Systemic Lupus Erythematosus Disease Activity Index

### Ethical considerations

All stages of the study consider the fundamental ethical principles underlying research involving human subjects and comply with international standards for scientific quality and ethics in the design, performance, registration, and reporting of intervention studies (Good Clinical Practices).

Before any procedure, the research subjects will sign a free and informed consent form (Additional file 1), and they will be informed that standard procedures were implemented to protect privacy and confidentiality of data collected and individual information generated by the study. For illiterate or visually impaired patients, the informed consent form will be read by the interviewer or the patient’s accompanying person. While informed consent is being taken, patients will be informed that they may be assigned to either the control group (routine treatment offered by the institution plus health counseling) or the intervention group (usual treatment offered by the institution plus the physician-pharmacist collaborative practice).

There are no known risks involved in participating in this study. However, in order to ensure and evaluate the study’s quality, scientific development, and ethical integrity, an external Data and Safety Monitoring Board (DSMB) will be created, consisting of health professionals not involved in the research project, including an epidemiologist, a pharmacist, and a statistician.

In case the study detects benefits resulting from the project, research subjects allocated to the control group will be ensured of follow-up by the pharmacist.

The study was approved by the Institutional Review Board of the Sérgio Arouca National School of Public Health/Oswaldo Cruz Foundation (Fiocruz), case file 845.155, and was registered in ClinicalTrials.gov under number NCT02330250 on 30 December 2014.

## Discussion

Increasing adherence to drug treatment has been identified as an important step in improving health outcomes for patients with chronic diseases, including SLE. Despite studies on the topic, there is still great uncertainty on how to improve adherence [5, 48]. Various studies have shown that the clinical pharmacist can play an important role in this scenario [49]. The complexity of therapy with multiple drugs with high potential for adverse events makes the pharmacist the element in the healthcare team with the greatest capacity to orient patients and answer treatment questions. As with other chronic diseases, low adherence in SLE is worrisome, since it has been associated with greater risk of hospitalization, renal injury, and mortality.

There are indications that the pharmacist’s intervention in this patient group increases medication adherence and can be incorporated into routine public healthcare services, with no need for special resources beyond the pharmacists themselves.

During data collection, patients treated at the study site requested orientation by the pharmacist independently of participation in the study and have been accompanied since then. Importantly, refusal to participate in the study has been rare. This demonstrates the potential of this approach for tackling the challenge of sustaining adherence levels consistent with the desired effectiveness in the clinical management of these patients.

Limitations in the present study include potential difficulty in establishing disease activity because this depends on immunological tests (serum complement level, anti-DNA antibody titer) that may not be available for SLEDAI measurements. However, the clinical components of SLEDAI, together with other laboratory items, are sufficient to assess disease activity [50]. Another potential limitation is patient absenteeism to scheduled visits, which might interfere with the detection of differences between groups. Nevertheless, the intervention itself is conceived to maximize treatment adherence, including compliance with scheduled visits. Another constraint is the general awareness about the study among patients and rheumatologists that may influence DTA (the Hawthorne effect) [51]. Lastly, reliability of measurements is known to influence the measurement of association. The MMAS-8 has previously shown good reliability. Moreover, the impact of limited reliability in this study will be reduced by taking the average of two measurements.

The current study aims to be the first to evaluate the effectiveness of PC for DTA in patients with SLE and seeks to produce information and knowledge that can help improve treatment in this group of patients.

Future reports will follow the CONSORT statement as well as its extension to non-pharmacological interventions (Additional file 2); [46, 52].

## Trial status

As of January 2015 the study had enrolled 86 % of the sample.

## Additional files

Additional file 1: Informed Consent Form. (DOCX 60 kb) Additional file 2: SPIRIT 2013: SPIRIT (Standard Protocol Items: Recommendations for Interventional Trials) checklist of the clinical trial protocol for effectiveness of pharmaceutical care for drug treatment adherence in patients with systemic lupus erythematosus. (DOC 142 kb)

## Abbreviations

ACR: American College of Rheumatology
ATC: Anatomical Therapeutic Chemical Classification System
CI: confidence interval
DNA: deoxyribonucleic acid
DSMB: Data and Safety Monitoring Board
DTA: drug treatment adherence
DURG: Drug Utilization Research Group
DUS: Drug Utilization Studies
Fiocruz: Oswaldo Cruz Foundation
ISN/RPS: International Society of Nephrology/Renal Pathology Society
MMAS-8: eight-item Morisky Medication Adherence Scale
PC: pharmaceutical care
SELENA/SLEDAI: Safety of Estrogens in Lupus Erythematosus National Assessment/Systemic Lupus Erythematosus Disease Activity Index
SF-36: Medical Outcomes Study 36-Item Short-Form Health Survey
SPIRIT: Standard Protocol Items: Recommendations for Interventional Trials
SLE: systemic lupus erythematosus
SLICC: The Systemic Lupus International Collaborating Clinics Group
WHO: World Health Organization
