# Erratum to: Effectiveness of pharmaceutical care for drug treatment adherence in patients with systemic lupus erythematosus in Rio de Janeiro, Brazil: study protocol for a randomized controlled trial

**DOI:** 10.1186/s13063-016-1766-6

**Published:** 2017-03-02

**Authors:** M. Oliveira-Santos

**Affiliations:** 1 Sergio Arouca National School of Public Health, Oswaldo Cruz Foundation, Rua Leopoldo Bulhões, 1480 Manguinhos, Rio de Janeiro Brazil; 2 Pedro Ernesto University Hospital, State University of Rio de Janeiro, Boulevard 28 de Setembro n° 77, Vila Isabel, Rio de Janeiro, Brazil

## Erratum

After publication of our article [1], it was brought to our attention that there was an error in reference 35. The correct reference is:

de Oliveira-Filho AD, Morisky DE, Neves SJ, Costa FA, de Lyra DP Jr. The 8-item Morisky Medication Adherence Scale: validation of a Brazilian-Portuguese version in hypertensive adults. Res Soc Adm Pharm. 2014;10(3):554–61.
