# Supplementary material for: Effectiveness of pharmaceutical care for drug treatment adherence in patients with systemic lupus erythematosus in Rio de Janeiro, Brazil: study protocol for a randomized controlled trial
Source: Trials. 2016 Apr 2;17:181. doi: 10.1186/s13063-016-1317-1 (PMC4818901; doi:10.1186/s13063-016-1317-1)
Supplement: Additional file 1: — Informed Consent Form. (DOCX 60 kb) [file 13063_2016_1317_MOESM1_ESM.docx]

**TERMO DE CONSENTIMENTO LIVRE E ESCLARECIDO**

**PROJETO: EFETIVIDADE DA ATENÇÃO FARMACÊUTICA NA ADESÃO AO TRATAMENTO NO LÚPUS ERITEMATOSO SISTÊMICO: UM ENSAIO CLÍNICO RANDOMIZADO**

Você está sendo convidado a participar da pesquisa: ***Efetividade da atenção farmacêutica na adesão ao tratamento no lúpus eritematoso sistêmico: um ensaio clínico randomizado,*** desenvolvida pela doutoranda Marise Oliveira dos Santos, da Escola Nacional de Saúde Pública Sergio Arouca da Fundação Oswaldo Cruz (ENSP/FIOCRUZ). A pesquisa tem como objetivo avaliar o efeito do acompanhamento farmacêutico na adesão ao tratamento dos pacientes com lúpus eritematoso sistêmico, atendidos no Ambulatório de Reumatologia do Hospital Universitário Pedro Ernesto (HUPE).

Você foi selecionado porque tem diagnóstico confirmado de lúpus eritematoso sistêmico e utiliza medicamentos corticosteroides e antimaláricos. Sua participação não é obrigatória e você tem plena autonomia para decidir se quer ou não participar. A qualquer momento você pode desistir de participar e retirar seu consentimento. Sua recusa não vai modificar em nada o seu relacionamento com seu médico nem com a equipe de saúde do Hospital Universitário Pedro Ernesto (HUPE) e sua participação na pesquisa não irá influenciar no seu acompanhamento médico regular (consultas e tratamento).

**_______________________ ________________________**

**Assinatura 1 assinatura 2**

A participação nesta pesquisa consistirá em responder algumas perguntas de como você usa os medicamentos prescritos por seu médico na consulta ambulatorial e sobre sua saúde. Serão consultadas também informações clínicas do seu prontuário médico.

Os pacientes estudados serão divididos em dois grupos. Os pacientes que forem destinados ao primeiro grupo serão acompanhados por um farmacêutico e no segundo grupo por um profissional de saúde que não será o farmacêutico. Você será destinado a um dos grupos de maneira aleatória (ou seja, por sorteio). O acompanhamento dos dois grupos ocorrerá no ambulatório de reumatologia do HUPE e consistirá em orientações sobre o tratamento de sua doença durante as suas consultas médicas ambulatoriais e uso de seus medicamentos. A diferença entre os grupos é que no grupo acompanhado pelo farmacêutico a orientação sobre o uso dos medicamentos será fornecida pelo médico e farmacêutico e no outro grupo somente pelo médico.

Não haverá despesas pessoais para você em qualquer fase do estudo, incluindo exames e consultas. Também não há compensação financeira relacionada à sua participação.

As informações obtidas através dessa pesquisa serão confidenciais e asseguramos o sigilo sobre sua participação. Cada paciente receberá uma identificação denominada neste estudo de “Nº AF” que não permitirá identificar seu nome. Os resultados serão divulgados sem identificação dos indivíduos. As entrevistas serão transcritas e armazenadas, em arquivos digitais, mas somente terão acesso às mesmas os pesquisadores envolvidos no projeto de pesquisa. Ao final da pesquisa, todo material será mantido em arquivo, por pelo menos 5 (cinco) anos, conforme as legislações brasileiras envolvendo as pesquisas (Resolução CNS/MS nº 466/2012 e complementares).

Esclarecemos que não são conhecidos riscos decorrentes da participação neste estudo. O benefício com sua participação é a produção de informações e conhecimentos que poderão contribuir para melhoria no tratamento dos pacientes com lúpus eritematoso sistêmico.

Você receberá uma via deste termo, cujas páginas serão rubricadas por você e pelo responsável pela pesquisa, nele consta também o telefone e o endereço institucional do pesquisador principal e do Comitê de Ética em Pesquisa (CEP). Você pode tirar suas dúvidas sobre a pesquisa e sua participação, agora ou a qualquer momento. O Comitê de Ética em Pesquisa é a instância que tem por objetivo defender os interesses dos participantes da pesquisa em sua integridade e dignidade e para contribuir no desenvolvimento da pesquisa dentro de padrões éticos.

___________________________________

MARISE OLIVEIRA DOS SANTOS

Contato com o(a) pesquisador(a) responsável: Contato com o CEP

Email.: [projetoadeles@gmail.com](mailto:projetoadeles@gmail.com) E-mail: [cep@ensp.fiocruz.br](mailto:cep@ensp.fiocruz.br)

Rua Leopoldo Bulhões, 1.480 - Sala 828 Endereço: Rua Leopoldo Bulhões, 1480 - Térreo Manguinhos - RJ /CEP. 21.041-210 Manguinhos - RJ/CEP: 21041- 210

Tel: (21) 2598 2685 Tel: (21) 2598 2863

Av. 28 de Setembro, 77 - Térreo

Vila Isabel - Rio de Janeiro – RJ / CEP: 20.511- 031

Tel: (21) 2868-8663

Declaro que entendi os objetivos, riscos e benefícios de minha participação na pesquisa e concordo em participar.

___________________________________________________________
